# PIpelle Prospective ENDOmetrial carcinoma (PIPENDO) study, pre-operative recognition of high risk endometrial carcinoma: a multicentre prospective cohort study

**DOI:** 10.1186/s12885-015-1487-3

**Published:** 2015-06-30

**Authors:** Nicole C. M. Visser, Johan Bulten, Anneke A. M. van der Wurff, Erik A. Boss, Carolien M. Bronkhorst, Harrie W. H. Feijen, Joke E. Haartsen, Hilde A. D. M. van Herk, Ineke M. de Kievit, Paul J. J. M. Klinkhamer, Brenda M. Pijlman, Marc P. M. L. Snijders, Ingrid Vandenput, M. Caroline Vos, Peter E. J. de Wit, Lonneke V. van de Poll-Franse, Leon F.A.G. Massuger, Johanna M. A. Pijnenborg

**Affiliations:** 1 Dept. Pathology, Radboud university medical centre, P.O. Box 9101, 6500 HB Nijmegen, The Netherlands; 2 Dept. Pathology, St Elisabeth Hospital, Tilburg, The Netherlands; 3 Dept. Obstetrics and Gynaecology, Maxima Medical Centre, Veldhoven and Eindhoven, The Netherlands; 4 Dept. Pathology, Jeroen Bosch Hospital, ′s-Hertogenbosch, The Netherlands; 5 Dept. Obstetrics and Gynaecology, Amphia Hospital, Breda, The Netherlands; 6 Dept. Obstetrics and Gynaecology, Elkerliek Hospital, Helmond, The Netherlands; 7 Dept. Pathology, Elkerliek Hospital, Helmond, The Netherlands; 8 Dept. Pathology, Canisius Wilhemina Hospital, Nijmegen, The Netherlands; 9 Dept. Pathology, PAMM, Eindhoven, The Netherlands; 10 Dept. Obstetrics and Gynaecology, Jeroen Bosch Hospital, ′s-Hertogenbosch, The Netherlands; 11 Dept. Obstetrics and Gynaecology, Canisius Wilhemina Hospital, Nijmegen, The Netherlands; 12 Dept. Obstetrics and Gynaecology, Catharina Hospital, Eindhoven, The Netherlands; 13 Dept. Obstetrics and Gynaecology, St Elisabeth Hospital, Tilburg, The Netherlands; 14 Dept. Pathology, Amphia Hospital, Breda, The Netherlands; 15 Dept. of Medical and Clinical Psychology, Tilburg University, Tilburg, The Netherlands; 16 Comprehensive Cancer Centre the Netherlands, Eindhoven, The Netherlands; 17 Dept. Obstetrics and Gynaecology, Radboud university medical centre, Nijmegen, The Netherlands; 18 Dept. Obstetrics and Gynaecology, TweeSteden Hospital, Tilburg, The Netherlands

**Keywords:** Endometrial carcinoma, Histological diagnosis, Endometrial sampling, Postmenopausal bleeding, Observational cohort study, Risk assessment

## Abstract

**Background:**

Endometrial carcinoma is the most common gynaecologic malignancy in industrialised countries and the incidence is still rising. Primary treatment is based on preoperative risk classification and consists in most cases of hysterectomy with bilateral salpingo-oophorectomy. In patients with serous and clear cell histology a complete surgical staging is mandatory. However, in routine clinical practice final histology regularly does not correspond with the preoperative histological diagnosis. This results in both over and under treatment.

**Methods/Design:**

The aim of this multicentre, prospective cohort study is to select a panel of prognostic biomarkers to improve preoperative diagnosis of endometrial carcinoma in order to identify those patients that need extended surgery and/or additional treatment. Additionally, we will determine whether incorporation of cervical cytology and comorbidity could improve this preoperative risk classification. All patients treated for endometrial carcinoma in the participating hospitals from September 2011 till December 2013 are included. Patient characteristics, as well as comorbidity are registered. Patients without preoperative histology, history of hysterectomy and/or endometrial carcinoma or no surgical treatment including hysterectomy are excluded. The preoperative histology and final pathology will be reviewed and compared by expert pathologists. Additional immunohistochemical analysis of IMP3, p53, ER, PR, MLH1, PTEN, beta-catenin, p16, Ki-67, stathmin, ARID1A and L1CAM will be performed. Preoperative histology will be compared with the final pathology results. Follow-up will be at least 24 months to determine risk factors for recurrence and outcome.

**Discussion:**

This study is designed to improve surgical treatment of endometrial carcinoma patients. A total of 432 endometrial carcinoma patients were enrolled between 2011 and 2013. Follow-up will be completed in 2015. Preoperative histology will be evaluated systematically and background endometrium will be classified. This is the first study incorporating immunohistochemistry, cervical cytology and comorbidity to define the optimal panel of prognostic biomarkers that contribute in clinical decision making in the management of endometrial carcinoma.

**Trial registration:**

Netherlands Trial Register number NTR3503

## Background

Endometrial carcinoma (EC) is the most common gynaecologic malignancy in the United States with approximately 52,630 diagnosed cases annually [1]. In the Netherlands the incidence is about 1900 women, with a mortality rate of 480 [2]. The incidence is still rising due to increased life expectancy and obesity as important risk factor [3]. Although the majority of patients are diagnosed at an early stage with a favourable prognosis, still around 20 % of patients die from the disease [4]. ECs are staged according to the 2009 Fédération Internationale de Gynécologie et d’Obstétrique (FIGO) classification. ECs are divided into two types. The majority of ECs are classified as type I and are related to unopposed oestrogenic stimulation resulting from obesity or exogenous hormone use and originate from hyperplastic endometrium. This tumour type is associated with early stage disease, endometrioid histology, and a favourable outcome after surgery [5]. In contrast, type II carcinomas are unrelated to oestrogenic stimulation and arise in a background of atrophic endometrium. Type II carcinomas are associated with advanced stage, high grade, non-endometrioid histology, and an overall a poor prognosis [5]. A recent study suggests the existence of a third type of EC characterised by low grade endometrioid endometrial carcinoma (EEC) and a background of atrophic endometrium [6]. This third type of EC may have a poorer prognosis when compared to type I carcinomas [6]. However, recently published data of The Cancer Genome Atlas (TCGA) Research Network, identified four subgroups of EC based on molecular classifiers such as *TP53*, *PTEN* and microsatellite instability [7]. This supports the need for adjusting the currently used classification.

### Primary treatment

Primary treatment is currently based on preoperative risk classification and consists of hysterectomy with bilateral salpingo-oophorectomy. In uterine papillary serous carcinoma (UPSC) and clear cell carcinoma (CCC) a complete surgical staging is mandatory because of the high risk of extra-uterine disease [8–10]. Although the presence of lymph node metastasis is an unfavourable predictor for disease specific survival, data of Kwon et al. demonstrated that high-risk uterine factors including high grade tumour type, deep myometrial invasion, and cervical stromal involvement are more significant determinants of survival in EC than pelvic-node status [11]. The current study focuses on diagnosis and preoperative risk assessment of patients with EC.

### Preoperative diagnosis

During the last decades dilatation and curettage (D&C) has been replaced by minimally invasive techniques for endometrial sampling in an outpatient setting. The amount of tissue obtained from endometrial sampling is relatively small and there can be different subtypes of EC in one tumour, making routine histological discrimination between EEC and a high grade, UPSC or CCC difficult. Moreover, in 30 % the amount of tissue obtained with outpatient endometrial sampling is insufficient for diagnosis [12]. Previous studies found discrepancy percentages between 15 and 40 %, including both grade and histological subtype [13–17]. When preoperative diagnosis was based on D&C or endometrial sampling, a preoperative diagnosis of grade 1 was concordant with the final diagnosis in 85 % of cases. However, high grade lesions were more frequently underestimated by endometrial sampling compared to D&C [18].

### Immunohistochemical analysis in preoperative endometrial sampling

Identification of a panel of immunohistochemical (IHC) markers may be helpful to establish a reliable preoperative risk classification. A brief summary of the selected markers that will be tested is given in Table 1. P53 immunopositivity is associated with non-endometrioid EC [19]. Negative IHC for oestrogen and progesterone receptors can predict lymph node metastasis and is associated with decreased survival [20]. Double negative hormone receptor status and p53 immunopositivity correlates with lymph node metastasis, high FIGO stage, non-endometrioid histology, high grade and poor prognosis [20]. Insulin-like growth factor II messenger RNA-binding protein 3 (IMP3) is a foetal protein not expressed in normal adult tissues. This oncoprotein plays an important role in tumour growth, migration and invasion. IMP3 could contribute to the preoperative identification of type II tumours, since it is more frequently expressed in UPSC and CCC when compared to EEC (resp. 78 %, 57 % and 15 %) [21]. A recent study showed that L1CAM is the best predicting prognostic factor in FIGO stage I, type I EC and superior to the standard used multifactor risk score (myometrial invasion, tumour grade and lymph space or vascular invasion) [22]. L1CAM immunohistochemistry can improve the identification of patients at risk for recurrent disease. However, all the mentioned biomarkers are lacking validation on pre-operative histological samples and are based on singles studies. Further research has to validate these promising results.

**Table 1:** Immunohistochemical analysis

Immunohistochemical marker		Results	Ref.
IMP3	Insulin-like growth factor II mRNA-binding protein 3	IMP3 is more frequently expressed in UPSC and CCC than in EEC (resp. 78 %, 57 % and 15 % of the tumours were positive).	[21]
P53		P53 is more expressed in non-endometrioid endometrial carcinomas than in EEC. Expression is also related to higher tumour grade.	[19]
ER and PR	Oestrogen and progesterone receptor	Negative receptors were associated with lymph node metastasis and decreased survival. ER and PR expression is lower in non-endometrioid endometrial carcinomas than in EEC.	[20, 34]
MLH1	MutL homolog 1	Loss of expression of mismatch repair proteins is seen in high grade EEC and not in UPSC and CCC. Loss of MLH1 expression is associated with longer survival.	[35, 36]
PTEN	Phosphatase and tensin homologue	PTEN positivity is more frequently found in UPSC than EEC.	[19]
Beta-catenin		Positive beta-catenin expression is associated with decreased stage, decreased grade and negative lymph node status	[37]
P16		Loss of p16 expression is significantly correlated with high FIGO stage and serous and clear cell histological subtype.	[38]
Ki-67		Higher Ki-67 expression is associated with higher tumour grade. UPSC and CCC show higher Ki-67 proliferation index than EEC.	[34]
Stathmin		Stathmin overexpression was associated with non-endometrioid histology, high grade and poor disease-specific survival.	[39]
ARID1A	AT-rich interactive domain 1A gene	Loss of ARID1A expression is significantly more frequent in high grade EEC compared to UPSC.	[40]
L1CAM	L1 cell adhesion molecule	L1CAM is associated with higher grade and non-endometrioid histology. Moreover, L1CAM positive EC have statistical significant poorer disease-free survival and overall survival.	[22]

*UPSC* = Uterine papillary serous carcinoma; *CCC* = Clear Cell Carcinoma; *EEC* = Endometrioid endometrial carcinoma; *EC* = endometrial carcinoma; *FIGO* = Fédération Internationale de Gynécologie et d’Obstétrique

### Preoperative diagnosis of EC in cervical cytology

The presence of endometrial cells in cervical cytology in postmenopausal women is strongly associated with endometrial pathology [23]. Abnormal cervical cytology is associated with extra-uterine disease in patients with UPSC and with cervical involvement in patients with EEC [24]. A combination of preoperative cervical cytology with endometrial sampling might better predict final histology and risk for extended disease. In a study of Kinde et al. DNA was extracted from cervical smears to detect genetic disorders present in EC [25]. The mutation profile found in the primary tumour was found in all of the cervical smears [25]. These results indicate that cervical cytology might be a reliable and minimal invasive source of material for detection of EC.

### Comorbidity and EC

The impact of comorbidity on cancer outcome has been underestimated for a long time. Recently published data demonstrated that EC patients with cardiovascular disease, previous malignancy and diabetes have a significantly decreased survival of 15–17 % compared to patients without comorbidity [26]. Additionally, patients with diabetes and EC have more comorbidities, higher body mass index (BMI) and higher FIGO stage, compared to those without diabetes [27]. There is also a significant increase in the risk of EC-specific mortality among women with diabetes [28]. Although obesity is a risk factor for development of EC, obesity seems not related to overall survival [29]. Yet, comorbidity has demonstrated to influence the outcome in EC [30].

In summary, the main challenging issue concerning clinical management of EC patients is underscored by the discordance between the preoperative risk classification of the tumour and the final surgical pathology. At the moment a subgroup of patients needs either a secondary surgical staging procedure or additional chemotherapy and/or radiation therapy. With the current study we want to select a panel of the most accurate biomarkers that can be used in daily practice for preoperative diagnosis of EC. This will aid in improving the concordance between preoperative and final histological diagnosis and thus prevent over and under treatment. Incorporating cervical cytology and comorbidity could potentially improve a proper risk classification in EC patients.

## Methods/Design

### Objective

#### Primary objective

To determine whether standardized evaluation of endometrial biopsies with additional immunohistochemical analysis, could predict final histological type, tumour grade and stage.

#### Secondary objective

To determine whether additional immunohistochemical analysis on endometrial biopsies could predict recurrence and disease free survival. Additionally, to determine whether incorporation of abnormal cervical cytology and comorbidity attributes to an improved risk classification.

### Study design

Multicentre, prospective cohort study in nine hospitals in the Netherlands. From September 1st 2011 till December 1st 2013 all patients treated for EC in participating hospitals are included. Patients without preoperative histology, history of hysterectomy and/or endometrial carcinoma or no surgical treatment including hysterectomy are excluded. Patient characteristics, as well as comorbidity (Charlson index), BMI, family history of hereditary syndromes (BRCA1/2, Lynch syndrome), postmenopausal status and parity are registered. Based on the present comorbidities, all patients are assigned a comorbidity score based on the Age-Adjusted Comorbidity-index as described by Charlson et al. [31], with EC being excluded from the scoring. Treatment and final pathological diagnosis are registered as well as occurrence of recurrent disease during at least 24 months follow-up.

### Tissue specimens

The endometrial biopsy or curettage on which the diagnosis of EC was made will be used for systematic evaluation by pathologists with special interest in gynaecologic pathology. Additional IHC analysis of IMP3, p53, ER, PR, MLH1, PTEN, beta-catenin, p16, Ki-67, stathmin, ARID1A and L1CAM will be performed (Table 1). Final pathology will be reviewed by the expert pathologists and compared with the preoperative histological diagnosis. The pathologists will be blinded for clinicopathological information and outcome.

### Methods

Tissue specimens are collected centrally at the department of Pathology, Radboud university medical centre in Nijmegen. Pre-operative samples will be evaluated on the amount of tissue (quantitatively and qualitatively), the presence of hyperplasia, atypia, endometrial intraepithelial carcinoma (EIC), invasive growth, background endometrium, tumour percentage and tumour type and grade. IHC staining will be performed on formalin-fixed, paraffin-embedded tissue of the pre-operative samples. IHC staining will be graded semiquantitatively by considering the percentage and intensity of the staining. A staining index will be calculated as the product of staining intensity and staining area.

### Statistical analysis

For the primary objective, results of endometrial biopsy and curettage will be compared with final pathology results. Both univariate as well as multivariate analysis will be performed to determine whether immunohistochemical markers contribute to prediction of final pathology. For the secondary objective we will also include abnormal cervical cytology and the Age-Adjusted Comorbidity-index as factors in univariate and multivariate analysis. In order to determine risk factors for recurrence Kaplan-Meier survival curves will be calculated to determine outcome after a follow-up time of 24 months. Statistical analysis will be performed using the Statistical and Data management package SPSS 20.0.

### Sample size calculation

Calculation of the sample size is based on the primary outcome variable of the study, which is high risk endometrial carcinoma. The smallest outcome group, in this case patients with high risk endometrial carcinoma, should be 10–20 times the amount of independent variables used.

Independent variables in the analyses will be: age (dichotomous), grade (1, 2 and 3) and the best predictive immunohistochemical markers. For the sample size calculation we assume to include six immunohistochemical markers in the multivariate analysis. Grade count as two variables because we use it as a trichotomous variable, which makes the total variables nine.

The amount of subjects in the smallest group therefore should lie between 90 and 180. However, as a rule of thumb, the amount of subjects should never be lower than 100.

With an expected high risk endometrial carcinoma rate of 25 % at least 400 patients with endometrial carcinoma should be included to include at least 100 patients with high risk endometrial carcinoma.

### Ethical considerations

This study is approved by the local medical ethical committee of the St Elisabeth Hospital Tilburg. According to the protocol “Code for Proper Use of Human Tissue”, all collected patient material will be coded, and patient name and date of birth are not entered into the database (Dutch Federation for Biomedical Scientific Societies, www.federa.org). We did not obtain written informed consent from patients because we use data anonymously according to the “Code for Proper Use of Human Tissue”. Included patients are informed about tissue and data use for scientific purpose in general and made no drawbacks.

## Discussion

A total of 432 EC patients from nine hospitals were collected between September 2011 and December 2013. The inclusion of patients has finished and we are now analysing the data. Follow-up will be completed in December 2015. The various histological subtypes of EC are all represented in this study group with 86 % EEC, 8 % UPSC and 2 % CCC based on hysterectomy evaluation. This is in line with percentages reported in the Netherlands Cancer Registry [32]. Clinicopathological characteristics are shown in Table 2. Atypical hyperplasia is diagnosed in 13 % of the preoperative endometrial samples, where final diagnosis on hysterectomy was EC.

**Table 2:** Clinicopathological characteristics of 432 women with endometrial carcinoma. Values are presented as median (range) or number (%)

Characteristics		
Age at primary surgery, years	66	(41–90)
Pre-operative histology^a^		
Office endometrial biopsy	311	(72.0)
Hysteroscopic biopsy	128	(29.6)
Curettage	75	(17.4)
Histological subtype^b^		
Endometrioid	370	(85.6)
Serous papillary	33	(7.6)
Clear cell	9	(2.1)
Mucinous adenocarcinoma	2	(0.5)
Carcinosarcoma	18	(4.2)
Histological grade^b^		
1	193	(44.7)
2	127	(29.4)
3	112	(25.9)
FIGO 2009 stage		
I	362	(83.8)
II	27	(6.3)
III	35	(8.1)
IV	8	(1.9)

^a^ 82 patients have more than one pre-operative histological sample

^b^ Unrevised classification based on hysterectomy specimen

We will determine if a diagnostic panel of IHC markers can improve the preoperative diagnosis for risk selection. This is the first study combining L1CAM with other markers to find the optimal panel of biomarkers for the preoperative diagnosis of EC. The additional value of immunohistochemical analysis in EC has been demonstrated in the large multicentre MoMaTEC trial [20], yet, this study focussed on predicting lymph node metastasis and prognosis in relation to treatment. Our focus is on preoperative risk classification with respect to histological type and tumour grade. Preoperative and final surgical pathology will be revised by expert pathologists. Furthermore, preoperative biopsy and curettage will be evaluated systematically, and compared with final histology. Additionally, background endometrium will be classified as: atrophic endometrium, hyperplastic endometrium, normal proliferative endometrium or indeterminate. Since, in a previous study on hysterectomy specimens, atrophic background endometrium was found to be an independent prognostic factor for patients with grade 1 EEC [6]. To date, no studies on the prognostic value of background endometrium in preoperative endometrial sampling are published.

Due to tumour heterogeneity and focal staining patterns, IHC on endometrial biopsies may not always be representative for the whole tumour. Most studies on IHC in endometrial carcinomas were performed on hysterectomy specimens. Yet, our clinical challenge is to select high risk tumour preoperatively on a limited amount of material. Our study design represents daily practice and with this study we will determine whether additional IHC analysis could predict final histology. Huang et al. reported comparable sensitivity for detecting high grade EC with Pipelle versus curettage [33]. The predictive value between endometrial biopsies and curettage might be different when IHC is applied, and hence influence outcome. To date, there are no studies on the influence of IHC on the difference in predictive value between biopsy and curettage. By using IHC the difference in the amount of material collected by biopsy and curettage might become relevant. Interestingly, incorporation of comorbidity in the preoperative risk classification has not been studied so far.

Summarizing, systematic preoperative evaluation of both tumour and patient characteristics could give maximal information and result in patient tailored treatment in patients with EC.

## Abbreviations

CCC: Clear cell carcinoma
D&C: Dilatation and curettage
EC: Endometrial carcinoma
EEC: Endometrioid endometrial carcinoma
EIC: Endometrial intraepithelial carcinoma
FIGO: Fédération Internationale de Gynécologie et d’Obstétrique
IHC: Immunohistochemical
UPSC: Uterine papillary serous carcinoma
